# Comparative Genomics of Smut Pathogens: Insights From Orphans and Positively Selected Genes Into Host Specialization

**DOI:** 10.3389/fmicb.2018.00660

**Published:** 2018-04-06

**Authors:** Juliana Benevenuto, Natalia S. Teixeira-Silva, Eiko E. Kuramae, Daniel Croll, Claudia B. Monteiro-Vitorello

**Affiliations:** ^1^Microbial Genetics Laboratory, Department of Genetics, University of São Paulo/Luiz de Queiroz College of Agriculture (USP/ESALQ), Piracicaba, Brazil; ^2^Department of Microbial Ecology, Netherlands Institute of Ecology (NIOO-KNAW), Wageningen, Netherlands; ^3^Laboratory of Evolutionary Genetics, Institute of Biology, University of Neuchâtel (UNINE), Neuchâtel, Switzerland

**Keywords:** Ustilaginaceae, host jump, effectors, orphan genes, positive selection

## Abstract

Host specialization is a key evolutionary process for the diversification and emergence of new pathogens. However, the molecular determinants of host range are poorly understood. Smut fungi are biotrophic pathogens that have distinct and narrow host ranges based on largely unknown genetic determinants. Hence, we aimed to expand comparative genomics analyses of smut fungi by including more species infecting different hosts and to define orphans and positively selected genes to gain further insights into the genetics basis of host specialization. We analyzed nine lineages of smut fungi isolated from eight crop and non-crop hosts: maize, barley, sugarcane, wheat, oats, *Zizania latifolia* (Manchurian rice), *Echinochloa colona* (a wild grass), and *Persicaria* sp. (a wild dicot plant). We assembled two new genomes: *Ustilago hordei* (strain Uhor01) isolated from oats and *U. tritici* (strain CBS 119.19) isolated from wheat. The smut genomes were of small sizes, ranging from 18.38 to 24.63 Mb. *U. hordei* species experienced genome expansions due to the proliferation of transposable elements and the amount of these elements varied among the two strains. Phylogenetic analysis confirmed that *Ustilago* is not a monophyletic genus and, furthermore, detected misclassification of the *U. tritici* specimen. The comparison between smut pathogens of crop and non-crop hosts did not reveal distinct signatures, suggesting that host domestication did not play a dominant role in shaping the evolution of smuts. We found that host specialization in smut fungi likely has a complex genetic basis: different functional categories were enriched in orphans and lineage-specific selected genes. The diversification and gain/loss of effector genes are probably the most important determinants of host specificity.

## Introduction

Host specialization is commonly found among plant pathogens. Specialist pathogens are favored in ecological contexts of restricted host species diversity, interspecific competition, and due to genetic trade-offs in adaptation to different hosts (Barrett et al., 2009; Johnson et al., 2009). Moreover, the co-evolutionary process itself is conducive to ever-increasing host specialization. The strong host selective pressure is likely to result in more specialized pathogen lineages over time and phylogenetically restricted host ranges (Gilbert and Webb, 2007; Johnson et al., 2009; Antonovics et al., 2013).

The intimate interaction between plants and specialist pathogens suggests that co-speciation should be common. However, host shifts/jumps rather than co-speciation are the main mode of pathogen speciation and a major route for disease emergence (Giraud et al., 2008, 2010; de Vienne et al., 2013; Choi and Thines, 2015). This raises intriguing questions such as how do specialized pathogens shift and specialize on a novel host, and which are the genetic determinants of host specificity. Different hosts have different defense mechanisms, biochemical composition, and associated microbiota to which pathogens must adapt to in order to be able to infect, colonize, feed and reproduce (Barrett and Heil, 2012; Haueisen and Stukenbrock, 2016). Hence, specialization to any specific host likely requires a different set of adaptations.

Many pathogens show extraordinary genome plasticity enabling the quick response to selection pressures imposed by a new host (Plissonneau et al., 2017). Analysis of host adaptation processes through comparative genomic studies showed that gene gain/loss, gene family expansion/contraction, and adaptive mutations were the most likely mechanisms across different pathosystems (Ma et al., 2010; Raffaele et al., 2010; Burmester et al., 2011; Baltrus et al., 2012; Kirzinger and Stavrinides, 2012; Grandaubert et al., 2015; Poppe et al., 2015; Yoshida et al., 2016; Zhong et al., 2016). Given the genetic specificity of each interaction, it is crucial to concurrently analyze a host-specialized species and its most closely related species. Adding more closely related species colonizing different hosts will likely reveal genomic differences reflecting adaptations to the host (Wollenberg and Schirawski, 2014).

Smut fungi are a relevant group of host specialized plant pathogens. Despite the growing interest in smut diseases as a threat to agriculture, edible delicacies, and biotechnological applications (Feldbrügge et al., 2013; Toh and Perlin, 2016), the genetic basis of host specialization in smut fungi remains largely unknown. Species from distinct subdivisions of the Basidiomycota are considered “smut” fungi. In this study, we refer to smut diseases within the Ustilaginaceae family, which comprises more than 600 species. Smut species infect hosts from many angiosperm clades. However, most of smut species are highly specialized on a single or a few host species, affecting mainly members of the Poaceae family (Begerow et al., 2004). Despite the restricted host range of smut pathogens, closely related pathogens do not infect sister host species. Such incongruence between host and pathogen phylogenies suggests that smut fungi become specialized mostly following host shifts within the Poaceae family (Begerow et al., 2004). The estimated divergence dates of four smut pathogens from agronomically important crops support the hypothesis that the host specialization evolved after the speciation of the host, but before the domestication of the host (Munkacsi et al., 2007).

Smut diseases are characterized by the production of a sooty dark brown mass of teliospores (Bakkeren et al., 2008; Morrow and Fraser, 2009). The life cycle comprises three genetically and morphologically distinct phases: diploid teliospores, haploid yeast like-cells and dikaryotic infective hypha (Piepenbring, 2009). Despite of their similarities, the mode of plant infection and symptom development vary among smut species. For example, *Ustilago maydis*, the causal agent of common smut of maize and teosinte, infects all aerial parts of the host plant (stems, leaves, tassels, and ears) and locally induces tumor formation (Bölker, 2001; Matei and Doehlemann, 2016); while most of smut species become systemic and the symptoms occur only in floral tissues (Piepenbring, 2009). The route of infection also varies among species, with some penetrating through the ovary, coleoptile, leaves, roots, or young buds. A common secondary symptom of many smut diseases is the hypertrophy of specific host organs, forming tumor-like galls. Other secondary symptoms described for some species are changes in inflorescence and branching architectures (Ghareeb et al., 2011), inducing the formation of multiple female inflorescences in *Sporisorium reilianum* infecting maize (Ghareeb et al., 2015) and tillering in *S. reilianum* infecting sorghum (Matheussen et al., 1991).

In order to investigate the genetic basis of host specialization, we performed a comparative genomics study of smut fungi, including seven previously available genome sequences. Additionally, we sequenced the genomes of two species isolated from wheat and oats to increase the scope of the host range. Hence, we compared a total of nine smut pathogens isolated from eight distinct hosts, including seven isolates from domesticated hosts (maize, barley, oats, wheat, sugarcane, *Zizania latifolia*) and two species infecting non-crop hosts (*Echinochloa colonum*, *Persicaria* sp.). The *Persicaria* sp. pathogen, *Melanopsichum pennsylvanicum*, is one of the few Ustilaginaceae smut species known to infect a dicot host (Sharma et al., 2014).

We compared the predicted effector content and the repertoire of plant cell wall degrading enzymes among smut lineages. Secreted effector proteins are key virulence factors in host interactions, acting to suppress host defenses and manipulate the physiology of the host (Kemen et al., 2015). Differences in effector repertoire were associated with the host range of different groups of pathogens (Kirzinger and Stavrinides, 2012; Feldbrügge et al., 2013; Rovenich et al., 2014). Plant cell wall-degrading enzymes play central roles in host penetration and nutrient acquisition during fungal infections. The arsenal of those enzymes also varies among fungi, reflecting their lifestyles and host preferences (King et al., 2011; Zhao et al., 2014). Zhao et al. (2014), for example, found that fungal pathogens of dicots often contain more pectinases than those infecting monocots. We also screened for genes with signatures of positive selection as different host species likely impose distinct selection pressures on the associated pathogen. Finally, we also analyzed evidence for species-specific genes as potential contributors to host specialization.

## Materials and Methods

### Strains, DNA Extraction and Sequencing

For genome sequencing, we selected *U. hordei* (strain Uhor01) isolated from an oats field in Southern Brazil and the *U. tritici* from CBS-KNAW Westerdijk Fungal Biodiversity Institute (strain CBS 119.19). Yeast-like cells were obtained from *U. hordei* teliospores according to Albert and Schenck (1996). Uhor01 is deposited under FioCruz Culture Collection accession number CFRVS 40435. For genomic DNA extractions, single colonies from both species were grown in YM liquid medium (0.3% yeast extract, 0.3% malt extract, 0.5% soybean peptone, 1% D-glucose), at 25°C overnight, in an orbital shaker at 250 rpm. Genomic DNA was extracted using the Genomic-tip 20G kit (Qiagen, Inc.), according to the manufacturer’s instructions for yeasts. A total of 10 μg of DNA of each sample was sent to the GCB facility at Duke University (United States), where a single large insert library (15–20 kb) was constructed and sequenced in one SMRT cell (P5-C3 chemistry) using the PacBio RS II (Pacific Biosciences, Inc.) sequencing platform. DNA from the same extraction was also used for Illumina paired-end library construction and sequencing using HiSeq2500 platform with 2 × 125 cycles at Center of Functional Genomics (ESALQ/USP, Brazil). About 10.4 Gb of Illumina and 1.6 Gb of Pacbio data were obtained for *U. hordei* and about 4.7 Gb of Illumina and 0.5 Gb of Pacbio data were obtained for *U. tritici*.

The genome and annotation files of *U. maydis*, *U. hordei*, *S. reilianum* were retrieved from MIPS^1^. The sequences of *U. esculenta*, *U. trichophora, S. scitamineum* were retrieved from NCBI^2^, and sequences of *M. pennsylvanicum* from Senckenberg Repository^3^. Among the genomes of *S. scitamineum* strains sequenced, we used the best assembly from SSC39B strain in our analyses (Taniguti et al., 2015), since low intraspecific variability was reported worldwide (Braithwaite et al., 2004; Raboin et al., 2007), and all strains were isolated from sugarcane hosts (Que et al., 2014; Dutheil et al., 2016). More information about the smut and outgroup species used in the present study are listed in **Table 1**.

**Table 1:** List of analyzed Ustilaginomycotina species, strains, and genomes assemblies.

	Abbreviations	Species	Strain	Host/source	Project number	Reference
**SMUTS**	*UhoO*	*Ustilago hordei*	Uhor01	*Avena sativa* (oats)	PRJNA393983	This work
	*Utri*	*Ustilago tritici syn. Tilletia tritici* (?)	CBS119.19	*Triticum* spp. (wheat)	PRJNA400640	This work
	*Umay*	*Ustilago maydis*	521	*Zea mays* (maize)	PRJNA1446	Kamper et al., 2006
	*UhoB*	*Ustilago hordei*	Uh4857-4	*Hordeum vulgare* (barley)	PRJEA79049	Laurie et al., 2012
	*Uesc*	*Ustilago esculenta*	MMT	*Zizania latifolia* (rice-relative)	PRJNA263330	Ye et al., 2017
	*Utcp*	*Ustilago trichophora*	RK089	*Echinochloa colona* (wild grass)	PRJNA316802	Zambanini et al., 2016
	*Srei*	*Sporisorium reilianum*	SRZ2	*Zea mays* (maize)	PRJNA64587	Schirawski et al., 2010
	*Ssci*	*Sporisorium scitamineum*	SSC39B	*Saccharum* spp. (sugarcane)	PRJNA275631	Taniguti et al., 2015
	*Mpen*	*Melanopsichium pennsylvanicum*	Mp4	*Persicaria* sp. (wild dicot plant)	PRJEB4565	Sharma et al., 2014
**NON-SMUTS**	*Mglo*	*Malassezia globosa*	CBS7966	Human	PRJNA18719	Xu et al., 2007
	*Msym*	*Malassezia sympodialis*	ATCC42132	Human	PRJEB417	Gioti et al., 2013
	*Pant*	*Moesziomyces antarcticus syn. Pseudozyma antarctica.*	JCM10317	Lake sediment	PRJNA302316	Morita et al., 2014
	*Paph*	*Moesziomyces aphidis syn. Pseudozyma aphidis*	DSM70725	Aphid excretions	PRJNA215967	Lorenz et al., 2014
	*Pbra*	*Kalmanozyma brasiliensis syn. Pseudozyma brasiliensis*	GHG001	Larva intestinal tract	PRJNA217085	de Castro Oliveira et al., 2013
	*Pflo*	*Anthracocystis flocculosa syn. Pseudozyma flocculosa*	PF-1	Leaf epiphyte associated with clover powdery mildew	PRJNA185206	Lefebvre et al., 2013
	*Phub*	*Pseudozyma hubeiensis*	SY62	Deep-sea cold-seep clam	PRJDB993	Konishi et al., 2013


**? misclassification detected in this work.**

### Genome Assembly and Synteny

We evaluated multiple approaches for the *de novo* assembly of the *U. hordei* and *U. tritici* genomes. A hybrid assembly using SPAdes v. 3.10.1 (Bankevich et al., 2012) and AHA from the SMRT Analysis 2.3.0 (Chin et al., 2013) produced the best assembly metrics for both species. SPAdes was run for Illumina reads with the parameters “-k 23,31,39,47,55,63,71,79,87,95” and “–careful”. AHA was run using the SPAdes assembly and PacBio reads with normal coverage parameters (default). To further improve the assembly, PBJelly from the PBSuite v15.8.24 (English et al., 2014) was used to fill intra-scaffold gaps in the AHA hybrid assembly through the alignment of long PacBio reads. For running PBJelly we set up the minimum number of gaps to start to cover with PacBio reads (–minGap = 1) and the blast aligner parameters (-minMatch 8 -minPctIdentity 70 -bestn 1 -nCandidates 20 -maxScore -500 -noSplitSubreads). Pilon v1.18 (Walker et al., 2014) with the parameters “–mingap 1” and “–fix bases, gaps” was also used to align Illumina short reads to the draft assembly in order to correct single base errors, minor mis-assemblies and to fill gaps. These Whole Genome Shotgun projects have been deposited at DDBJ/ENA/GenBank under the accessions NSHH00000000 and NSDP00000000. The versions described in this paper are versions NSHH01000000 and NSDP01000000.

Pairwise genome dot plots were generated using the R-package DECIPHER (Wright, 2016). Sequence homology was defined using *k*-mer exact nucleotide matches. Hits were further chained into blocks of synteny with default parameters.

### Gene Prediction and Annotation

Genes in the genomes of *U. hordei*, *U. tritici*, *U. esculenta*, and *U. trichophora* were predicted using Augustus v.2.5.5 (Stanke and Morgenstern, 2005). Protein sequences of *U. maydis*, *U. hordei*, and *S. scitamineum* were used as extrinsic sources of gene structure evidence to improve sensitivity of gene predictions. For this, exonerate v.2.2.0 (Slater and Birney, 2005) was used to generate hints from protein sequence alignments (protein2genome option). Then, Augustus v.2.5.5 was run using the hints file, complete gene model, and *U. maydis* as reference species.

All predicted proteomes were annotated using InterProScan v.5.19 (Jones et al., 2014). Pfam protein families, InterPro domains, gene ontology (GO) classification, and metabolic pathways were recovered (Supplementary File S1). The predicted secretome was defined by the presence of a signal peptide and absence of any transmembrane domain, using Phobius v.1.01 (Käll et al., 2004) and SignalP v4.1 (Bendtsen et al., 2004). EffectorP was used to predict the effector repertoire from the predicted secretome based on machine learning (Sperschneider et al., 2016). Characterized effectors in smut species were screened for orthologs and tblastn was used to search for homologous regions in smut genomes. Previously available transcriptomic data (Zhang et al., 2013; Taniguti et al., 2015; Ye et al., 2017) were used to validate mispredicted candidate effector genes using CLC Genomics Workbench V8.01 (CLC Bio).

The proteomes were also screened for CAZymes (carbohydrate active enzymes) (Lombard et al., 2013) using Hmmscan from the HMMER v3.1b2 package^4^ and the dbCAN HMM profile database (Yin et al., 2012). The hmmscan-parser script provided by dbCAN was used to select significant matches. Searches for lipases were also performed with Hmmscan using the “Lipase Engineering Database” (Fischer and Pleiss, 2003). Putative peptidases were identified by using batch BLAST at the MEROPS server (Rawlings et al., 2012). The secondary metabolite biosynthesis clusters were predicted by AntiSMASH web version 4.0.0 (Medema et al., 2011).

Distribution of euKaryotic Orthologous Group (KOG) terms were performed for protein sets using the BLAST search online tool against the eggNOG 4.0 database^5^. One-tailed Fisher’s exact test for KOG enrichment were performed for orphan and positively selected gene sets using the KOGMWU R package (Dixon et al., 2015).

### Repeats and Transposable Elements

*De novo* and homology-based identification of repeats were performed using the RepeatModeler pipeline. A combined repeat library was constructed concatenating the RepBase library (release of August 2015) with the *de novo* repeat family predictions. The combined repeat library was used as input for RepeatMasker^6^.

### Orthologous Groups

Orthologous and paralogous groups among the nine genomes were determined using OrthoMCL with default parameters: BLASTp *e*-value cutoff of 1e-5, percent match cutoff of 50, and inflation index of 1.5 (Li et al., 2003). The output of OrthoMCL was parsed to separate core and unique clusters, singletons, single-copy, and one-to-one orthologous genes. Orphan genes included singletons (genes not assigned to any OrthoMCL group) and unique clusters (cluster of paralogs unique to one species). For the phylogenetic tree reconstruction, OrthoMCL was also performed including the genome of additional *Ustilaginomycotina* fungi: *Malassezia globosa*, *Malassezia sympodialis*, *Pseudozyma antarctica*, *P. aphidis*, *P. brasiliensis*, *P. flocculosa*, *P. hubeiensis* (for references see **Table 1**).

### Phylogenetic Tree

A total of 1,776 one-to-one orthologous proteins from 16 genomes (including non-smut species) were aligned using MUSCLE v.3.6 (Edgar, 2004). Gblocks v.0.91b (Castresana, 2000) was used to remove all gaps (-b5 = *n*) and blocks with length smaller than 5 (-b4 = 5) in each alignment. After Gblocks filtering, protein alignments smaller than 100 amino acids were excluded. A total of 1,637 protein alignments were retained and concatenated for a total 624,996 amino acid positions. The best-fit amino acid substitution model for the data was obtained using ProtTest v.3.4.2 (Darriba et al., 2011). The model of LG+I+G+F was selected based on the likelihood and Bayesian criteria. A maximum likelihood phylogenetic tree was constructed using RAxML v.8.2.8 (Stamatakis, 2014) with 100 rapid bootstrap replicates. ASTRAL v.4.10.8 (Sayyari and Mirarab, 2016) was used to score the RAxML super matrix tree by each individual gene tree to provide the fraction of the induced quartet trees that is present in the super matrix tree. We compared the phylogenomic tree with the widely used ITS (Internal Transcribed Spacer)-based tree using NCBI accessions (see Supplementary File S1).

### Positive Selection

A total of 4,374 protein-coding sequences with one-to-one orthologs among the nine smut species were aligned with the codon-aware aligner pal2nal v.14 (Suyama et al., 2006) and gaps were removed from the final alignment. After filtering, 4,195 sequence alignments were retained. The protein sequences were used to build a smut phylogenetic tree using the methods described above. The ETE3-evol tool (Huerta-Cepas et al., 2016) was used to automate codeml analysis (Yang, 2007). Sites-specific and branch-sites models at each lineage were applied. For the site-specific analysis, assuming variable selective pressures among amino acid sites, we performed likelihood ratio tests (LRTs) between two pairs of models: M2 (selection) against M1 (neutral) and M8 (beta&ω) against M7 (beta), according to Yang et al. (2000). Bayes Empirical Bayes (BEB) was employed to infer which sites in the alignment are under positive selection (≥0.95). For branch-site analyses, assuming variable selective pressures among sites and branches in the phylogeny, we specified each lineage as foreground branch at each round of analysis. For *U. hordei* lineages, we also consider the species branch (named *UhoOB*) as the foreground branch. LRTs was performed between the models bsA (neutral/relaxation) and bsA1 (positive selection) and BEB was used for detect significant sites (≥0.95), according to Zhang et al. (2005).

### SNP Detection Between *U. hordei* Strains

The scaffolds from the genome assembly of the *U. hordei* strain isolated from oats were aligned to the reference genome of *U. hordei* strain isolated from barley using the NUCmer module from MUMmer v3.0 (Kurtz et al., 2004). We used the repeat-masked genomes to avoid repetitive regions. To find the set of single nucleotide polymorphisms (SNPs) in the alignment, we used the module “show-snps” with -Clr option to select only SNPs in uniquely aligned sequence.

## Results

### Genome Assembly of *U. hordei* and *U. tritici*

**FIGURE 1:**
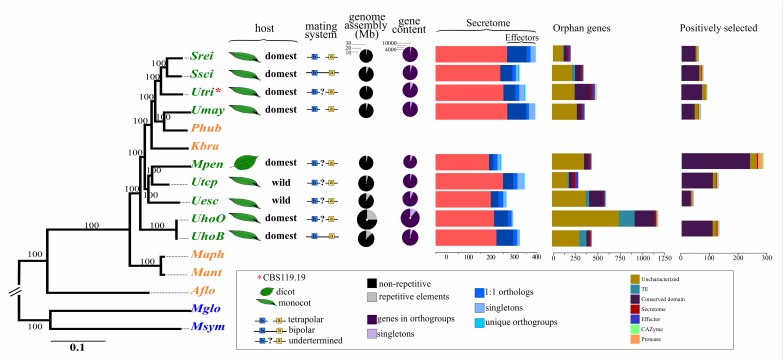
Maximum likelihood phylogenomic tree of *Ustilagomycotina* species based on 1,637 one-to-one orthologous genes, host information, and genomic features of smut fungi. The scientific names were abbreviated according to **Table 1**. The colors assigned to each species distinguish plant pathogens (green), human pathogens (blue), and species from distinct environmental niches (orange). Double bars at the tree root indicate out of scale.

We assembled the genomes of two smut species infecting important crops (*U. hordei* from oats and *U. tritici* from wheat) using a combination of Illumina and PacBio reads. The *de novo* assemblies resulted in a genome size of 18.63 Mb assembled in 73 contigs ( = 500 bp) for *U. tritici* and 24.63 Mb assembled in 2,200 contigs ( = 500 bp) for *U. hordei*. The assembly of *U. tritici* had a higher degree of contiguity (N_50_ 610 kb) than *U. hordei* (N_50_ 40 kb). A total of 7,892 and 6,776 protein-coding genes were predicted in the *U. hordei* and *U. tritici* genomes, respectively (Supplementary File S1). For both species, the two mating-type loci (*a* and *b*) were each located on different scaffolds (**Figure 1**).

### Phylogenomics

To reconstruct the phylogeny of smut fungi we included seven additional species from the subphylum Ustilagomycotina. One-to-one protein orthologs were concatenated and used to build a super-matrix tree. The super-matrix tree had a quartet support of 64.80% (i.e., 64.80% of all quartet trees induced from gene trees were present in the super-matrix tree).

The phylogenomic tree showed that the genus *Ustilago* was not monophyletic, clustering with members of the *Sporisorium Melanopsichum, Pseudozyma*, and *Kalmanozyma* genera (**Figure 1**). The dicot-infecting species, *M. pennsylvanicum*, was closely related to the monocot-infecting pathogens in the phylogenetic tree. The phylogeny of the smut fungi also did not separate pathogens according to the wild or domesticated status of their hosts. *U. hordei* was placed as the earliest diverging species among the analyzed smut fungi.

Surprisingly, however, was the phylogenetic positioning of *U. tritici* (former *Tilletia tritici* (Bjerk.) G. Winter, 1874) acquired from the CBS-KNAW culture collection under the accession CBS 119.19 (**Figure 1**). The CBS-KNAW *U. tritici* strain was placed close to *Sporisorium* species and clustered apart from *U. tritici* and *Tilletia* species in the ITS-based tree using NCBI accessions (Supplementary File S1), suggesting misclassification. Henceforward, we will refer to this strain by its CBS accession number to avoid misinterpretation.

### *U. hordei* Strains Comparison

**FIGURE 2:**
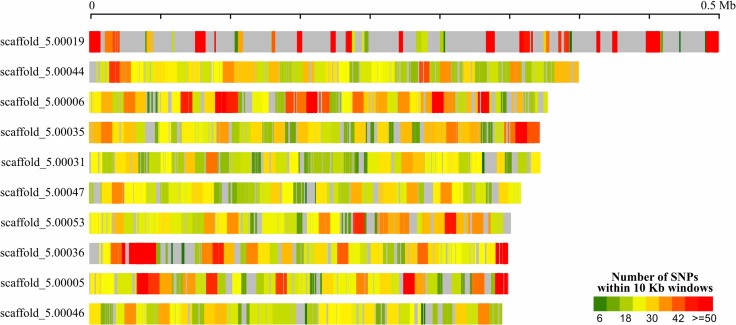
Distribution of SNPs in 10 Kb windows across the 10 largest *Ustilago hordei* (barley) scaffolds. Repeat-masked genomes were used in NUCmer alignment. Repeat-masked or SNP-poor regions are shown in gray.

A total of 17,454,837 bp (70.83%) of the *U. hordei* genome from oats aligned to the *U. hordei* strain from barley, not taking into account the repetitive regions that represents 25.12% of its genome. Within the aligned regions, 54,935 SNPs were detected which are scattered throughout the genome, although in distinct density (**Figure 2**).

### Genomic Synteny

**FIGURE 3:**
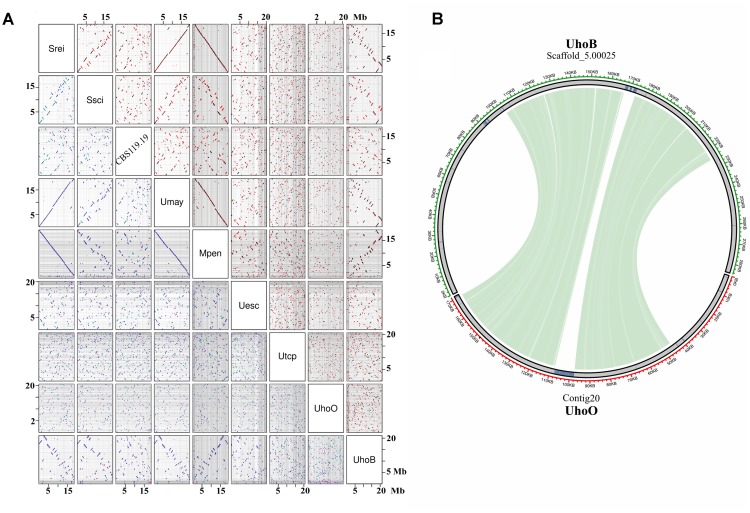
Synteny between smut genomes. **(A)** Pairwise dotplot showing the syntenic blocks between smut genomes. The scientific names were abbreviated according to **Table 1**. Axes represent the concatenation of repeat-masked chromosomes or scaffolds of each species (Mb scale). Grid lines indicate the boundaries between scaffolds. Dots in the upper diagonal correspond to regions of sequence similarity, where black color represents forward matches and red color represents reverse-complement matches. Dots in the lower panel are color-coded by the alignment score, with green meaning higher score and blue/purple lower score. **(B)** Circos-plot of *U. hordei* (oats) largest scaffold showing the syntenic region in *U. hordei* (barley) scaffold. Green lines connecting scaffolds correspond to blastn searches using repeat-masked scaffolds. Repetitive regions are shown in blue.

Pairwise dotplot sequence comparisons showed more evident syntenic relationship among high quality assembled genomes, since fragmented genomes result in many tiny syntenic blocks. Interestingly, conservation of long-range synteny was observed between more distantly related species, such as *S. reilianum* – *U. maydis* – *M. pennsylvanicum* (**Figure 3A**). More extensive chromosomal rearrangements were observed between closely related species, such as *S. reilianum* – *S. scitamineum* – CBS119.19, suggesting that these events occurred after the species diverged from the last common ancestor. Translocations and inversions also occurred at mating-type harboring scaffolds (Supplementary File S2). Despite of the fragmented assemblies and transposable elements-rich scaffolds, small syntenic regions were also observed between *U. hordei* strains (**Figure 3B**).

### Comparative and Functional Genomics of Smut Fungi

**FIGURE 4:**
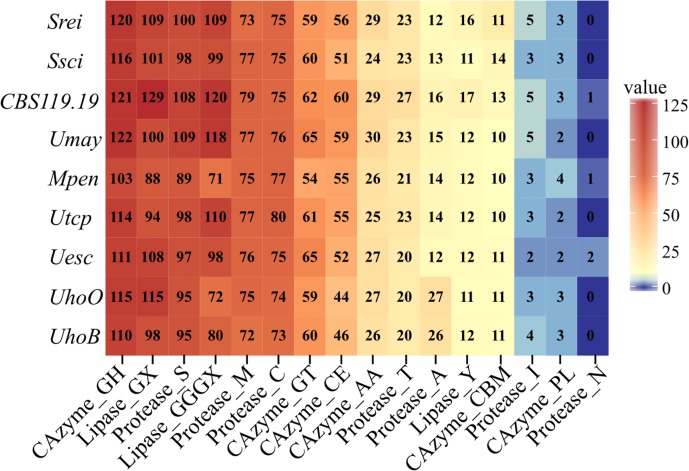
Heatmap of CAZymes, proteases and lipases classes. The numbers of enzyme categories in each genome are shown. Classes and modules of CAZymes include: GHs, glycoside hydrolases; CEs, carbohydrate esterases; CBMs, carbohydrate-binding modules; GTs, glycosyl transferases; PLs, polysaccharide lyases; AAs, auxiliary activities). Proteases are classified by the catalytic type of the proteolytic enzymes: A, aspartic; C, Cysteine; M, metallo; N, asparagine; S, serine; T, threonine; and also inhibitors of peptidases (I). Lipases are classified into three classes on the basis of the oxyanion hole: GX, GGGX, and Y. The scientific names were abbreviated according to **Table 1**.

*Ustilago hordei* species showed the largest genomes among the smut fungi, which ranged from 18.38 Mb in *S. reilianum* to 24.63 Mb in *U. hordei* (oats) (**Table 2** and **Figure 1**). The larger genome size in *U. hordei* species was also accompanied by an increase in the repetitive elements content. In particular, the content in transposable elements ranged from 0.61% in the genome of CBS119.19 strain to 23.93% in *U. hordei* (oats). The predicted gene repertoire varied from 6,280 genes in *M. pennsylvanicum* to 7,892 in *U. hordei* (oats). *M. pennsylvanicum* also encoded the smallest number of secreted protein (291) and predicted effectors (55), while *S. reilianum* had the largest secretome (443) and effector content (127). The total number and the diversity of sub-categories of CAZyme, protease, and lipase domains were similar among smut species (**Figure 4**). *U. hordei* species were an exception, because there was an expansion of the peptidase family A11A (*Copia* transposon peptidase) compared to the other species (Supplementary File S3). Around ten secondary metabolite biosynthesis clusters were identified in all smut genomes (**Table 2**). All species have at least one cluster encoding for putative terpene synthase (TS), non-ribosomal peptide synthase (NRPS), and type 1 polyketide synthases (t1PKS). Only *U. trichophora* presented a hybrid cluster of NRPS-Indole-t1PKS.

**Table 2:** Genomic statistics of smut fungi.

Genomic statistics	*Srei*	*Ssci*	CBS119.19	*Umay*	*Mpen*	*Uesc*	*Utcp*	*UhoO*	*UhoB*
**Assembly**									
Total assembly size (Mb)	18.38	19.95	18.63	19.64	19.23	20.19	20.68	24.63	21.15
Average base coverage	29×	500×	278×	10×	339×	139×	na	487×	25×
Number of contigs (> =500 bp)	45	26	73	27	435	298	215	2200	713
N50 (bp)	772,363	875,830	610,801	884,984	121,670	403,507	179,640	39,442	307,727
Largest contig (bp)	2,448,206	2,009,762	1,118,949	2,476,501	690,500	1,882,320	637,988	171,399	542,606
GC-content (%)	59.87	55.16	57.08	54.03	50.90	54.42	53.06	51.60	52.16
**Coding sequences**									
Number of genes	6,776	6,677	6,776	6,784	6,280	6,773	6,499	7,891	7,111
Single-copy genes	6,159	6,080	6,335	6,175	5,791	6,057	5,925	6,896	6,500
Co-orthologs groups	6,214	6,007	6,055	6,111	5,591	5,808	5,890	6,319	6,351
Genes into the groups	6,492	6,382	6,297	6,454	5,853	6,287	6,239	7,026	6,717
Paralogs	617	597	441	609	489	716	574	995	611
Unique groups	8	19	8	12	7	32	17	82	21
Singletons	183	295	479	330	427	486	260	865	394
Total of orphan genes	201	356	500	367	447	607	299	1186	447
**Repeat sequences (%)**									
Simple/tandem repeats	2.04	1.76	1.75	1.68	1.59	2.12	2.06	1.59	1.60
Interspersed repeats/TEs	0.66	4.10	0.61	2.47	2.30	7.54	2.15	23.93	11.68
Total of bases masked	2.68	5.85	2.34	4.13	3.88	9.65	4.18	25.12	13.21
**Secretome**									
Predicted secretome	443	371	397	441	291	314	394	343	373
Predicted effectors (EffectorP)	127	85	97	124	55	70	96	85	104
Predicted effectors (size/Cys)	47	33	42	58	20	29	42	50	63
**Secondary metabolic clusters**									
Terpene	2	3	3	2	2	2	3	1	1
Nrps	2	3	2	3	1	2	2	2	2
T1pks	1	1	1	1	1	1	1	1	1
Nrps-Indole-T1pks	0	0	0	0	0	0	1	0	0
Other	5	6	6	7	5	5	5	5	5
**Positively selected genes**									
Branch-sites model	60	76	90	67	286	41	128	131^∗^	131^∗^


**The species names were abbreviated according to **Table 1**. ^∗^Species branch selected (UhoOB); na = not available.**

**FIGURE 5:**
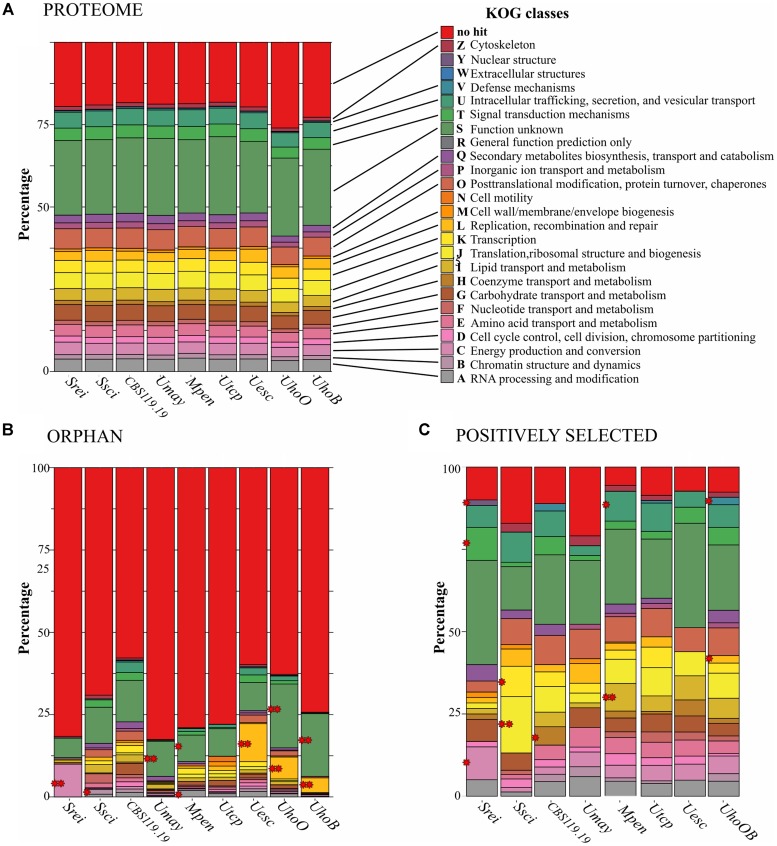
Diversity and distribution of KOG categories among smut protein-encoding gene sets. **(A)** KOG categories in the genomes of each smut lineage. **(B,C)** Distribution of KOG categories in orphan and positively selected genes, respectively. Significant enriched categories by Fisher’s exact test are indicated by red asterisks (^∗^*p*-value < 0.05, ^∗∗^*p*-adjusted < 0.05). The scientific names were abbreviated according to **Table 1**. *UhoOB* represents the *U. hordei* species branch marked for positive selection analyses.

We compared the predicted proteome of nine smut species and found 7,187 orthologous clusters (orthogroups) using OrthoMCL (Supplementary File S4). Out of those, 4,706 were shared among all species, wherein 4,374 were one-to-one orthologs. The average of protein identity varied from 72% among orthologs of *U. hordei* and *U. maydis* to 98% among orthologs of the two *U. hordei* strains (Supplementary File S4). The closest species regarding protein sequence identity were *S. reilianum* and *S. scitamineum*, as also observed by the phylogenomic tree. The general content of functional categories was very similar among smut species (**Figure 5A**).

*Sporisorium reilianum* had the smallest set of orphan genes (201) and *U. hordei* (oats) the largest (1,186) (**Figure 1**). Most of the species-specific encoded proteins were uncharacterized (lacking a conserved domain). Transposases and reverse transcriptases were frequent among orphan proteins, mainly in *U. hordei* proteomes. Some predicted effectors (ranging from 7 in *U. hordei* (oats) to 32 in *U. trichophora*) were also species-specific. Among the orphan proteins with a conserved domain, we found enzymes acting in primary and secondary metabolic pathways, proteins associated with transcriptional regulation, signaling, cell cycle control, morphogenesis, and stress response (Supplementary File S5). Functional enrichment analysis using KOG terminology showed that terms related to replication, recombination and repair were overrepresented in *U. esculenta* and *U. hordei*; RNA processing and modification was overrepresented in *M. pennsylvanicum;* energy production and conversion was overrepresented in *S. reilianum*; chromatin structure and dynamics was overrepresented in *S. scitamineum;* secondary metabolites biosynthesis, transport, and catabolism was overrepresented in *U. trichophora* (**Figure 5B**).

**FIGURE 6:**
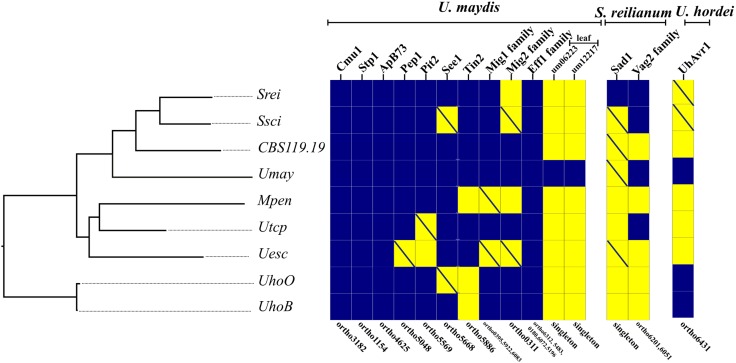
Presence (blue squares) and absence (yellow squares) of genes or gene families encoding known effectors in *U. maydis*, *S. reilianum*, and *U. hordei* based on OrthoMCL groups. The OrthoMCL groups are indicated in the lower part of the figure. Crossed yellow squares indicate that a similar genic region is present in the genome by tblastn search. The species are ordered according to their phylogenetic relationship. The scientific names were abbreviated according to **Table 1**.

Effectors characterized in *U. maydis*, *S. reilianum*, and *U. hordei* (found on barley) were screened for orthologs in the other species. We identified some effectors that were present in all proteomes, including Cmu1 (*Chorismate mutase 1*), Stp1 (*Stop after penetration 1*), ApB73 (*Apathogenic in B73*), and members of the *Eff1* family. Homology searches by tblastn identified putative orthologs of some effectors (Supplementary File S6). Therefore, orthologs of Pep1 (*Protein essential during penetration-1*), See1 (*Seedling efficient effector1*), and members of the Mig1 (*Maize-induced gene 1*) family were also detected in all genomes. Additional effectors were only found in a subset of species (**Figure 6**). The leaf-specific effector candidates, *um06223* and *um12217*, were present only in *U. maydis*. The effector Sad1 (*Suppressor of apical dominance 1*) was specific to *S. reilianum* using the automated annotation procedure, but then recovered in the genomes of *S. scitamineum*, CBS119.19, *U. maydis*, and *U. esculenta*. The genomic region coding for Pit2 (*Protein involved in tumors 2*) was also identified in *U. trichophora*. Although the 14 highly conserved residues of Pit2 protein sequence were detected in *U. trichophora* sequence, the signal peptide is missing. It remains to be established whether Pit2 is secreted using a non-conventional pathway and its functional role if any in *U. trichophora*. Phylogenetic trees and protein identity matrices of effectors are provided in Supplementary File S6.

We also checked the expression of mispredicted candidate effector genes based on previously available transcriptomic data (Zhang et al., 2013; Taniguti et al., 2015; Ye et al., 2017). Candidate genes identified by tblastn encoding See1 and UhAvr1 were found expressed in *S. scitamineum* both *in vitro* and *in planta*, and Sad1 only *in planta.* Genes encoding Pep1 and Sad1 were expressed in *U. esculenta* also in *in vitro* and *in planta*, and the gene encoding UhAvr1 in *S. reilianum* was expressed *in vitro* (Supplementary File S6). The transcriptomic data provided additional evidence for the presence of functional orthologs.

### Positively Selected Genes

Using site-specific models, we found significant evidence for positive selection at individual sites in 31 genes (Supplementary File S7). These genes were identified by both M2/M1 and M8/M7 model comparisons. Out of the 31 genes, three encoded proteins of unknown function. Interesting conserved domains included those associated with the regulation of transcription, such as the *bE* mating-type-specific homeodomain; synthesis of lipids, such as diacylglycerol acyltransferase domain; and response to environmental stresses, such as cyclophilin domain.

Branch-site models were also used to identify sites evolving under episodic selection. For *U. hordei* lineages analyzed individually, only nine genes in each lineage were detected to be under selection (Supplementary File S7). Therefore, we considered the *U. hordei* species branch for comparison. Positively selected sites exclusively found in one species varied from 41 in *U. esculenta* to 286 in *M. pennsylvanicum* and included genes encoding CAZymes and proteases. Among those, *M. pennsylvanicum* showed the highest number, with five CAZymes from esterases families and 15 protease genes under positive selection (**Figure 1**). Among the positively selected genes, there were also genes encoding enzymes acting on primary and secondary metabolism, proteins associated with regulation of cell cycle and morphogenesis, signaling, response to stress (Supplementary File S7). Functional enrichment analysis showed significant overrepresentation of distinct categories among positively selected gene sets: “Lipid Transport and Metabolism” and “Intracellular Trafficking, Secretion, and Vesicular Transport” for *M. pennsylvanicum;* “Energy Production and Conversion,” “Signal Transduction Mechanisms,” and “Nuclear Structure” for *S. reilianum*; “Translation, Ribosomal Structure and Biogenesis” and “Transcription” for *S. scitamineum*; “Cell motility” and “Defense Mechanisms” for *U. hordei* species; and “Coenzyme Transport and Metabolism” for CBS119.19 strain (**Figure 5C**).

## Discussion

In this study, we extended the comparative genomic analyses of Ustilaginaceae smut fungi by using seven genomes available. In order to increase the host range survey, we additionally sequenced and assembled two genomes: *U. hordei* (Uhor01 strain) and a new specimen also belonging to Ustilaginaceae isolated from wheat (CBS119.19 strain). The similarities in cell biology and lifestyle among the nine smut species was reflected in the fact that more than 65% of orthologs groups were shared among species. Most of the orthologous genes were detected as one-to-one orthologs. The species shared also a similar content of KOG functional categories. However, among those genes we found significant evidence of episodic positive selection. Moreover, sets of orphan genes were detected for each species. Hence, each genome offered a particular repertoire of genes that can be related to host-specialization. We are aware that it is difficult to distinguish the genetic changes that directly contribute to the host specialization from those that were a consequence of the divergence after host specialization, but some insights are discussed.

### Complex Evolution of Smut Fungi: Taxonomic and Gene Tree Discordances

The phylogenomic tree based on a distance super-matrix approach showed that the genus *Ustilago* remained polyphyletic, despite of many taxonomic revisions into to the Ustilaginaceae family have been recently proposed (McTaggart et al., 2012, 2016; Wang et al., 2015). Another discordance detected was regarding the classification of the *U. tritici* strain used in this work. The strain was placed close to *Sporisorium* species unlike other smut phylogenies based on multiple genes (Stoll et al., 2003; Begerow et al., 2006; McTaggart et al., 2012). The *U. tritici* taxonomic designation for the CBS 119.19 strain was based on phenotypic data from the time of accession at CBS-KNAW collection (Gerard Verkleij, personal communication) and, therefore, requires revision.

The decomposition of gene trees in quartets showed some additional phylogenetic conflicts. Dutheil et al. (2016) argued for incomplete lineage sorting as a source of phylogenetic incongruence among smut species, but undetected paralogy, recombination, natural selection and hybridization events could also have caused the discordant gene tree topologies. Kellner et al. (2011) detected a high potential for hybridization in some extant smut species. Hybridization is recognized as a major force in generate new host specificities (Stukenbrock et al., 2012; Depotter et al., 2016; Menardo et al., 2016) and hybridizations may well have occurred in the evolutionary history of smut fungi. Determining the processes causing conflicting signals among gene trees has the potential to better elucidate the evolutionary history of smut fungi. A comparison between the divergence at syntenic and rearranged regions will be also interesting to show if rearrangements had protected from interspecific gene flow by suppressing recombination (Rieseberg, 2001; Stukenbrock, 2013).

### Expansion of Repetitive Elements in *U. hordei* Isolates

The smut pathogens sequenced so far have compact genomes depleted of paralogs and repetitive DNA. *U. hordei* is an exception and clearly experienced a genome expansion by containing more protein-coding genes and repetitive elements. Dutheil et al. (2016) speculated that the activity of transposons in the *U. hordei* genome is under less stringent control and that active transposons have translocated some candidate effector genes. The sequencing of a second *U. hordei* strain herein supports the hypothesis of active transposable elements by showing an even greater content of repetitive DNA than the previously sequenced strain.

In many cases, the genomic plasticity and rapid evolution of pathogens have been associated with the activity of transposons (Wöstemeyer and Kreibich, 2002; Raffaele and Kamoun, 2012; Castanera et al., 2016). An example of this activity was found in *U. hordei*, where virulent and avirulent isolates on Hannchen barley cultivar differed for an insertion of a transposon-derived sequences in the promoter region of the *UhAvr1* effector gene (Ali et al., 2014). The insertion modulated the gene expression and likely the recognition by the host resistant protein Ruh1. Both *U. hordei* scaffolds harboring the *UhAvr1* gene are enriched in repetitive elements. However, comparison between the genomic context of *UhAvr1* gene in the oats isolate was not conclusive, since the gene is very close to the scaffold terminus (Supplementary File S2).

In addition to the difference in the content of transposable elements that can affect the genomic context of effectors and cause chromosomal rearrangements, we also identified SNPs in non-repetitive regions between the *U. hordei* strains. The number of SNPs detected between *U. hordei* strains is almost four times higher than between two *S. scitamineum* strains infecting sugarcane detected by Taniguti et al. (2015) and both species have bipolar mating system, which indicates that selfing is the primary mode of reproduction. However, most of the SNPs do not cause missense mutations, since the average of protein sequence identity among *U. hordei* strains were around 98%. All these genomic differences may be contributing for the ability to infect different hosts and even for the emergence of *formae speciales*. However, further experiments to determine the specificity of these interactions, a better genome assembly for detecting chromosomal rearrangements, and population genomics studies encompassing more barley and oat isolates can provide more evidences of their divergence and detect the ongoing genome evolution via transposable elements activity.

### The Content of Plant Cell-Wall Degrading Enzymes Seems to be Unrelated With Host Specialization in Smut Pathogens

The distribution of CAZymes, proteases, and lipases categories were similar among the nine smut pathogens analyzed herein. The amount of CAZymes in smut species is in agreement to what is reported for other biotrophic fungi (Zhao et al., 2014). Biotrophic fungi tend to have fewer CAZymes than hemibiotrophs and necrotrophs, causing minimal damages to their hosts (Kim et al., 2016). As other biotrophic pathogens, smut fungi also lack the glycoside hydrolase family 6 (GH6) which has a well-known cellulase activity for plant cell wall degradation (Zhao et al., 2014). However, we detected other gene families encoding cellulose, hemicellulose, pectin, and cutin degrading enzymes in smut genomes.

We found no expansion in pectinase content in *M. pennsylvanicum* in relation to Poaceae-smut pathogens. This is in disagreement with the previous finding of a dicot-related expansion by Zhao et al. (2014). However, *M. pennsylvanicum* has species-specific and positively selected CAZymes and proteases that may have contributed to the dicot-host adaptation.

The most discrepant pattern among the analyzed enzymes was in the aspartic peptidase A11A family that was only expanded in *U. hordei* genomes. The A11A family contains endopeptidases encoded by retrotransposons that act on polyprotein processing, adding to the evidence of genome expansion by transposons in *U. hordei* genomes.

### The Acquisition of an Optimal Effector Gene Repertoire

Using a machine learning approach, we identified a variable number of predicted effector genes among smut species. The smallest secretome and effector repertoire of *M. pennsylvanicum* were already identified by Sharma et al. (2014) who proposed that gene losses were the hallmark of the host jump event to a dicot host. The *U. esculenta* genome harbored the second lowest secretome and effector gene repertoire among smut species. We suggest that relaxed selection pressure may have led to the reduced effector gene content in this species. Infected *Z. latifolia* results in an edible smut gall and *U. esculenta* has been propagated together with the host through asexual rhizome by human activities (Chung and Tzeng, 2004). As *U. esculenta* spends its entire life cycle in the host plant and has been artificially maintained *in planta* over centuries, some effectors may be no longer essential since there is no need to re-infect the host. The long-standing effects of artificial selection in this pathosystem was in-depthly explored by Ye et al. (2017), who also reported the absence of genes coding for surface sensors and amino acid biosynthesis pathways in *U. esculenta* genome.

Among the few functionally characterized effectors in smut pathogens, some were shared among all smut species and might constitute core virulence factors for the establishment of the disease or enhancing pathogen fitness. Using the tblastn search associated with the transcriptomic data available, we were able to identify some missing effectors by the automatic gene prediction. Hence, all smuts species analyzed have orthologs of c*mu1*, *stp1*, *apB73, pep1*, *see1*, and members of the *mig1* and *eff1* family of effectors. Cmu1, Stp1, and Pep1 are known as defense-suppressing virulence effectors (Djamei et al., 2011; Hemetsberger et al., 2012; Liang, 2012) and overcoming the basal host defense responses is likely needed for all smut species.

The effector See1 was characterized in *U. maydis*-maize interaction and is required for tumor formation in leaf cells (Redkar et al., 2015a). Despite the organ-specific role of See1 and the fact that *U. maydis* is an exception among smut pathogen by its ability to locally induce tumor formation in leaves, *see1* orthologs were present in all other smut genomes. Nonetheless, Redkar et al. (2015b) showed that the *U. hordei see1* does not functionally complement the deletion mutant of *U. maydis*. Hence, *see1* orthologs may have a distinct role in other smut fungi, since transcriptomic data showed that the coding gene is expressed during *S. scitamineum* and *U. esculenta* respective interactions. However, besides *see1*, other leaf-induced candidate effector genes (*um06223* and *um12217*) (Schilling et al., 2014) were specific of *U. maydis* genome, suggesting a role in host adaptation and specific symptom development. Their functional roles await further investigation.

Another interesting example is the effector gene *SAD1* of *S. reilianum*, whose orthologs in the *S. scitamineum* and *U. esculenta* genomes were identified in this work. The *S. reilianum* SAD1 effector alters the inflorescence branching architecture of maize plants by inducing loss of apical dominance (Ghareeb et al., 2015), which could also be responsible for the tillering symptom reported for smutted sugarcane (Sundar et al., 2012) and *Z. latifolia* (Yan et al., 2013). Using previously available transcriptomic data (Taniguti et al., 2015; Ye et al., 2017), we also showed that *SAD1* is expressed during the respective host–pathogen interactions.

Other characterized smut effectors showed distinct pattern of presence/absence. Such effectors have been shown in other studies to be related to particular symptoms of the pathosystem, interact differently with host molecules, and/or have minor effects on virulence (Basse et al., 2000, 2002; Khrunyk et al., 2010; Ali et al., 2014; Schilling et al., 2014; Tanaka et al., 2014; Ghareeb et al., 2015; Redkar et al., 2015a,b; Zhao, 2015; Stirnberg and Djamei, 2016; Lanver et al., 2017). Moreover, although some effectors have orthologs, in some cases their protein sequences were poorly conserved and failures in cross-species complementation assays were observed in other studies (Redkar et al., 2015b; Stirnberg and Djamei, 2016). As effectors are subject to strong selection pressure to evade coevolving plant defenses, it is also likely that some effectors diverged to an extent that they are no longer recognized as orthologs by our criteria. Sets of lineage-specific candidate effectors were detected by our comparative study and we suggest that these genes are good candidates for further characterization in regards to their role in virulence and host specificity.

### Orphan and Positively Selected Genes: Potential Metabolic Versatility, Life-Cycle Orchestration, and Host Molecule Recognition

By increasing the number of species in the comparative genomics analyses, we found a smaller number of orphan genes than in the comparison of four genomes performed by Sharma et al. (2014) and Taniguti et al. (2015). This indicates that some previously identified orphan genes were in fact shared among closely related species. The majority of the orphan genes encoded proteins without well-characterized domains. Otherwise, most genes with signatures of positive selection encoded conserved domains. We found that different KOG categories were enriched among the positively selected gene sets, providing evidence for lineage-specific functional diversification.

We found few gene clusters encoding enzymes for secondary metabolite biosynthesis in smut genomes and, to our knowledge, no phytotoxin production was so far reported for smut fungi. However, *U. trichophora* genome presented a unique NRPS-Indole-t1PKS hybrid cluster and secondary metabolism pathways were overrepresented among its orphan genes. Besides toxins, secondary metabolites can have several roles in pathogenesis, such as effectors (manipulating gene expression and host physiology), siderophores, protection against biotic and abiotic factors (Pusztahelyi et al., 2015). Hence, the secondary metabolism is an interesting target to further explore in the *U. trichophora*–*E. colona* interaction.

Some orphan and positively selected genes were also associated with primary metabolic pathways, potentially generating metabolic versatility. For instance, orphans and positively selected genes in *S. reilianum* were enriched in “energy production and conversion” enzymes, such as reductases, oxidases, and dehydrogenases. Such enzymes participate in the oxidative phosphorylation pathway, but also in the oxidative stress (Marcet-Houben et al., 2009). The oxidative stress can have several roles during fungal-plant interactions (Breitenbach et al., 2015). Interestingly, Ghareeb et al. (2011) showed that *S. reilianum*-colonized inflorescences had a higher level of reactive oxygen species than in healthy maize inflorescences, which were specifically accumulated around fungal hyphae. Hence, the differentiation of these enzymes in *S. reilianum* may be related to the strong oxidative stress faced by the pathogen or with the production of reactive oxygen species during the symptom development.

*M. pennsylvanicum*, the dicot pathogen, had a much larger number of genes under positive selection than monocot-infecting species. Among the enriched classes were “Lipid transport and metabolism” and “Intracellular trafficking, secretion, and vesicular transport.” Differences in the lipid metabolism can be associated with the capacity to utilize distinct carbon sources from the dicot host or also with the production of signaling molecules. Studies of lipid signaling networks in pathogenic fungi have been shown roles in trigger and mediate cell cycle and growth, as well as virulence factors to counteract host defenses (Singh and Poeta, 2011). The intracellular trafficking in filamentous fungi is required for polarity establishment, hyphal growth, and/or virulence (Wang and Shen, 2011). The endocytic process is involved in signal perception, nutrient uptake, and ion homeostasis; while the secretory process delivers effectors and cell wall-degrading enzymes into the plant apoplast.

For *U. hordei* and *U. esculenta*, the enriched class detected among the orphan genes was “Replication, recombination, and repair,” since these species have the highest content of repetitive elements, particularly retrotransposons, that were considered orphan genes.

Other potentially affected pathways by orphan and positively selected genes were signaling, regulation of transcription, cell-cycle control, and morphogenesis. These genes may orchestrate the infection and development of the pathogens in their respective host. Smut species present distinct sporulation time and penetrate at distinct sites (Chung and Tzeng, 2004; Brefort et al., 2009; Ghareeb et al., 2011; Schaker et al., 2016; Marques et al., 2017). We speculate that some of the encoded proteins may act on the perception of different host molecules as a signal for penetration or induction of fungal sporogenesis, as these stages are also related to cell cycle control and morphogenesis. Other interesting proteins among these sets are those with peptide signal. For example, genes encoding copies of the potentially secreted RlpA-like protein (fungal expansin-like proteins) are also within the orphan list. Expansins are cell wall-loosening proteins without enzymatic activity and also an adhesion facilitator for fungal cells to plant cells by binding hydrophobic surfaces (Nikolaidis et al., 2014). As each species has divergent versions of this protein, it may be associated with specific host interaction.

Using site-specific models, positive selection acting on specific codons were found in 31 genes. Among those, we identified positive selection in the mating-type *bE* locus. In smut fungi, the *bE* locus encodes for the component of the heterodimeric bE/bW homeodomain transcription factor that triggers filamentous growth and pathogenicity after compatible yeast-like cell recognition and fusion (Bakkeren et al., 2008). Positive selection at *bE* sites could promote reproductive isolation among species by non-dimerization with *bW.* However, the selection signature identified herein may also be due to biased allele sampling among the sequenced genomes. Selection at specific-sites was also identified in a gene encoding a putative diacylglycerol acyltransferase enzyme that acts in the final step of triacylglycerol (TG) synthesis. TG is a storage lipid which serves as energy reservoir, source of signaling molecules, and substrate for membrane biogenesis (Liu et al., 2012). The TG biosynthesis pathway is conserved in all living organisms; however, sequence motifs of diacylglycerol acyltransferase are not conserved (Turchetto-Zolet et al., 2011). In *S. scitamineum*, the gene encoding for this enzyme was upregulated during sporogenesis (Taniguti et al., 2015), which may be related to the accumulation of lipid droplets in teliospores that will serve as a source of energy during germination (Marques et al., 2017). This enzyme was also associated with pathogenicity in the broad host range pathogen *Colletotrichum gloeosporioides* (Sharma et al., 2016). The significance of the selected sites for functional differences remains to be explored.

## Conclusion

In summary, our comparative genomic study provided further insights on smut host-specificity and symptoms development. In addition to sequencing and characterize two new genomes (from CBS 119.19 strain and *U. hordei* isolate from oats), we also brought new knowledge to less studied smut species (*M. pennsylvanicum*, *U. trichophora*, and *U. esculenta*). We identified lineage-specific sets of orphans and positively selected genes enriched for different functional categories, highlighting genes that have a potential role in host–pathogen interaction. The presence of distinct effector repertoires, with some being detected exclusively in each genome, is emphasized as the most likely important determinants of host specificity. Therefore, we provided good candidate genes for further functional characterization in different smut species. A comparative transcriptomic profile will also achieve additional insights, since differences in host specificity can also be due to distinct expression pattern of orthologs. Moreover, the comparison of *U. hordei* isolates herein showed the ongoing activity of transposable elements, with variable amounts predicted between the two strains. A population genomic study in *U. hordei* is also promising to reveal the extent of the divergence among barley and oat isolates.

## Author Contributions

JB analyzed the data. JB, DC, and CM-V designed the analyses and wrote the manuscript. EK and NT-S contributed to the purchase and *in vitro* growth of the isolates and commented on and edited the manuscript. CM-V and DC supervised the research.

## Conflict of Interest Statement

The authors declare that the research was conducted in the absence of any commercial or financial relationships that could be construed as a potential conflict of interest.

## Footnotes

1 http://mips.helmholtz-muenchen.de/

2 http://www.ncbi.nlm.nih.gov

3 http://dx.doi.org/10.12761/SGN.2014.3

4 http://hmmer.org/

5 http://eggnog.embl.de

6 http://www.repeatmasker.org/
