# Supplementary material for: Comparative Genomics of Smut Pathogens: Insights From Orphans and Positively Selected Genes Into Host Specialization
Source: Front Microbiol. 2018 Apr 6;9:660. doi: 10.3389/fmicb.2018.00660 (PMC5897528; doi:10.3389/fmicb.2018.00660)
Supplement: Supplementary file 7 [file Image_1.pdf]

Supplementary File 2 – Mating-type Synteny

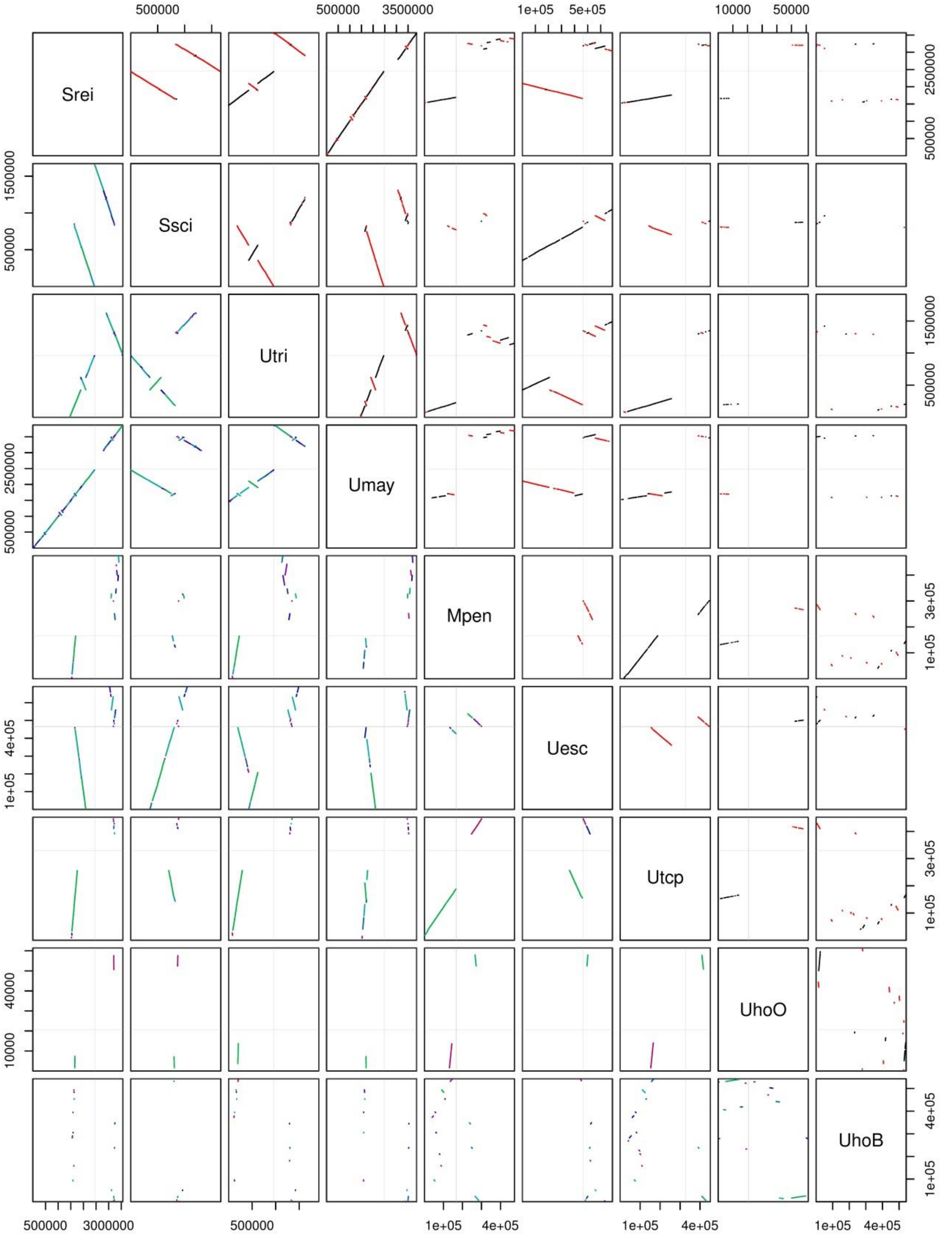

Figure 1. Pairwise dotplot synteny between mating-type harboring scaffolds. The scientific names were abbreviated according to Table 1. Axes represent the concatenation of scaffolds of each species (bp scale). Grid lines indicate the boundaries between scaffolds. Dots in the upper diagonal correspond to regions of sequence similarity, where black color represents forward matches and red color represents reverse-complement matches. Dots in the lower panel are color-coded by the alignment score, with green meaning higher score and blue/purple lower score.

UhAVR1 genomic context among *U. hordei* strains

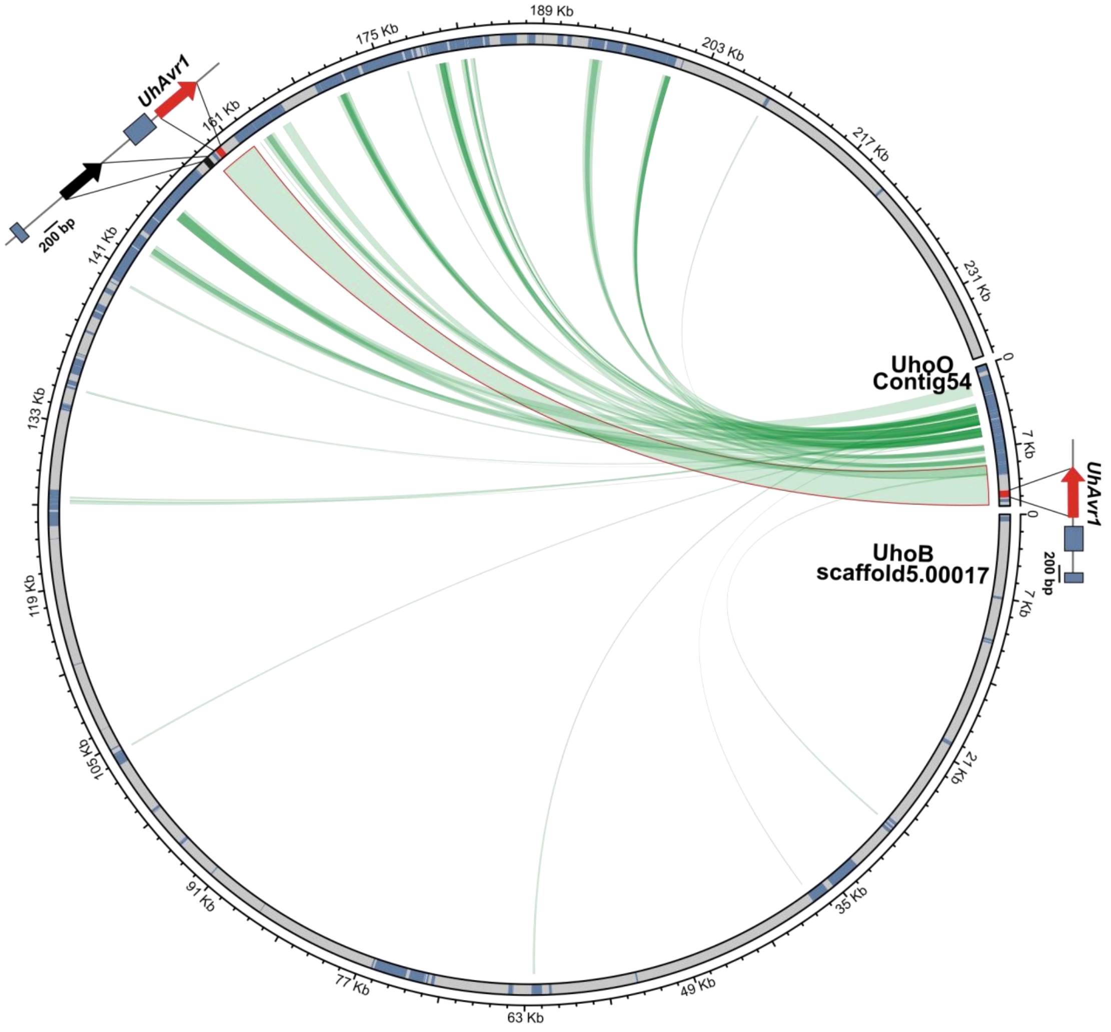

Figure 2. Circus plot of scaffolds harboring *UhAvr1* gene in *U. hordei* isolates (*UhoB* and *UhoO*). The *UhAvr1* gene is shown in red and its paralog is shown in black. Transposable elements are shown in blue. Green lines connecting scaffolds correspond to blastn searches.
